# C-756-08. Using AI to Develop Burn-Specific Resources for Pediatric Patients and Visitors

**DOI:** 10.1093/jbcr/irag033.052

**Published:** 2026-04-06

**Authors:** Stacey Richerbach, Karen J Richey, Kevin N Foster

**Affiliations:** Diane & Bruce Halle Arizona Burn Center - Valleywise Health, Phoenix, AZ; Diane & Bruce Halle Arizona Burn Center - Valleywise Health, Phoenix, AZ; Diane & Bruce Halle Arizona Burn Center - Valleywise Health, Phoenix, AZ

## Abstract

**Introduction:**

Pediatric burn patients, as well as their families and visitors are at risk of experiencing fear, anxiety, and distress. Distraction-based interventions, such as coloring, may decrease perceived pain and improve emotional well-being. Historically, our center inconsistently provided generic, non-medical coloring books, which may be less effective than materials reflecting the burn care environment. Burn staff employed artificial intelligence (AI) to create a burn- and facility-specific coloring book and expand coloring’s therapeutic and educational utility. This quality improvement project aimed to evaluate whether AI could effectively generate such resources for children.

**Methods:**

A Burn Nurse Specialist designed the coloring book using photos of patient care areas, wound care supplies, therapy equipment, and members of the burn team. Images were uploaded into an AI platform. A standardized prompt was used to produce age-appropriate image outlines for coloring and words of encouragement were added to select pages to support positive coping. Content was validated by burn staff. Fifty prototype books were printed and distributed with individual-use crayons at inpatient stations and the outpatient registration desk. Use frequency was tracked, and both staff and caregivers provided qualitative feedback on distraction, cooperation, and emotional response. Data collection occurred over a 4-month period.

**Results:**

Books required regular restocking, underscoring demand. In the first three months, distribution averaged 20 books per month. Fifty books were used in month four, reflecting a 150% increase in use. Staff noted improved cooperation during waiting periods and procedures. Caregivers reported comfort, familiarity with the care environment, and positive reinforcement. No safety concerns arose.

**Conclusions:**

AI enables rapid conversion of real, non-identifiable images into safe, cartoon-style outlines, producing personalized therapeutic tools. AI-assisted coloring books represent a low-cost intervention that fosters pediatric comfort and cooperation while enhancing caregiver and staff satisfaction. Incorporating familiar imagery not only promotes reassurance and engagement but also serves as a practical educational tool within the burn care setting. This project demonstrates the value of integrating AI into pediatric burn care resources. Future directions include expansion of procedure-specific content, digital adaptations, and quantitative study of long-term effects on anxiety reduction.

**Applicability of Research to Practice:**

Generic distraction tools may not adequately prepare children for burn care. By integrating de-identified images from the burn environment, a burn-specific coloring book may enhance both education and comfort.

**Funding for the Study:**

N/A.

